# DISSEMINATED CYSTICERCOSIS WITH HUGE MUSCLE HYPERTROPHY

**DOI:** 10.4103/0019-5154.48987

**Published:** 2009

**Authors:** Debabrata Bandyopadhyay, Sumit Sen

**Affiliations:** *From the Department of Dermatogy, R.G. Kar Medical College and Hospital, Kolkata, West Bengal, India*

**Keywords:** *Cysticercosis*, *muscle hypertrophy*, *subcutaneous nodules*

## Abstract

Cysticercosis is caused by cysticercus cellulose, which is the larva of *Taenia solium*, the pork tapeworm. The larvae are carried in the blood stream after penetrating the walls of the alimentary tract and they lodge in different tissues like the skin, skeletal muscles, brain, fundus and heart, to cause disseminated cysticercosis. Cases of disseminated cysticercosis have rarely been reported in the literature. They may inhabit the muscles and cause muscular hypertrophy, which, at times, may assume gross proportions. Morbidity is usually caused by the involvement of the central nervous system or the eyes.

## Introduction

Cysticercosis results in humans, after ingestion of eggs of *Taenia solium*, usually from close contact with a tapeworm carrier. The larval form can invade many tissues in humans, to cause disseminated cysticercosis. Generalized symmetrical pseudohypertrophy of muscles, in such conditions, has previously been reported only in twelve cases.

## Case Report

**Figure 1:**
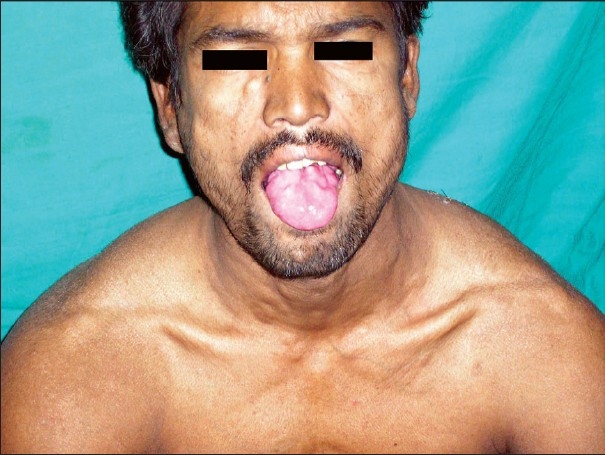
Cysticercus nodules on tongue

**Figure 2:**
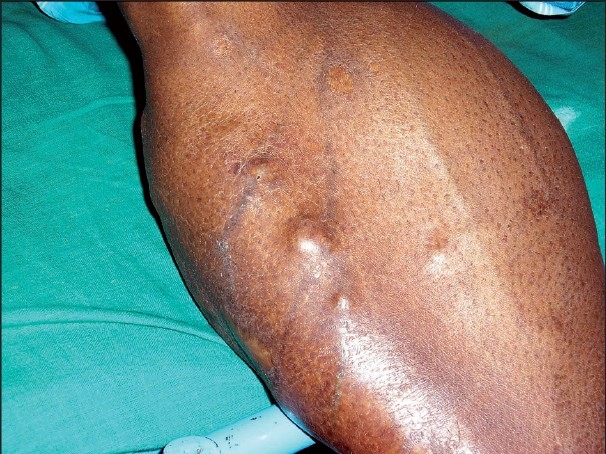
Venous prominence over hypertrophied calf muscle with a nodule

A male patient, laborer by profession and aged about 40 years, was referred from the Medicine department to the Dermatology department. The patient complained of vertigo and tingling sensation of both lower limbs. Huge swelling of both calf muscles and hypertrophy of shoulder joint muscles of both sides had gradually developed over the last five years. There were pea sized, soft, painless, well demarcated multiple nodules scattered over his body and tongue [Figure 1, Figure 2]. He gave a history of ingestion of pork.

**Figure 3:**
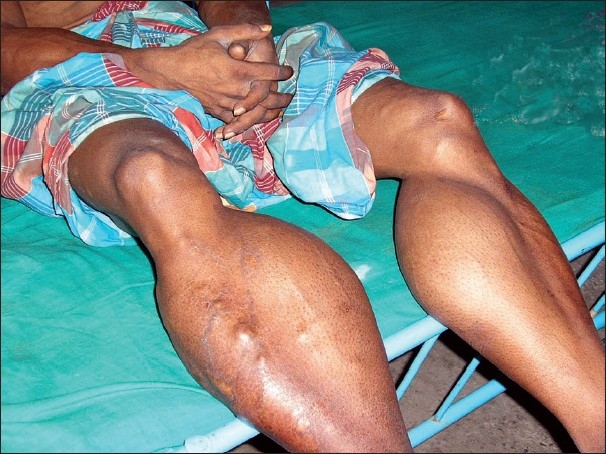
Cysticercosis with gross hypertrophy of calf muscles

Swelling started from the calf muscles [Figure 3], progressed to affect the deltoids, trapezius, triceps and biceps, and the gluteal muscles of both sides, and slowly grew to very large size. These muscles were not tender. The patient was unable to walk unaided at this time.

Venous prominence was clearly discernible over the affected muscles [Figure 2]. The person complained of blurring of vision and an ophthalmoscopy revealed an annular white scar over the disc of the right eye. The patient was disturbed by heaviness of the head, which was accompanied by frequent focal seizures. There was no vomiting or fever. Sleep, bladder and bowel habits were normal. He was conscious, oriented and alert, though anxious. Investigations revealed the following: hemoglobin = 11.4 gm/dl, white blood cell count = 2750, neutrophils = 55%, lymphocytes = 38%, eosinophils = 4%, monocytes = 3%. Microscopic examination of three stool samples over three consecutive days showed some cysts of *E.histolytica*, but no eggs or larvae of *Taenia solium*. Fasting blood sugar, urea and creatinine were normal. Creatinine phosphokinase was 60 units. Human immunodeficiency virus (HIV) testing was non reactive.

Ultrasonography (USG) of the calf muscles revealed hugely enlarged calf muscles, with evidence of multiple intramuscular cystic lesions. There was no evidence of any increased vascularity.

**Figure 4:**
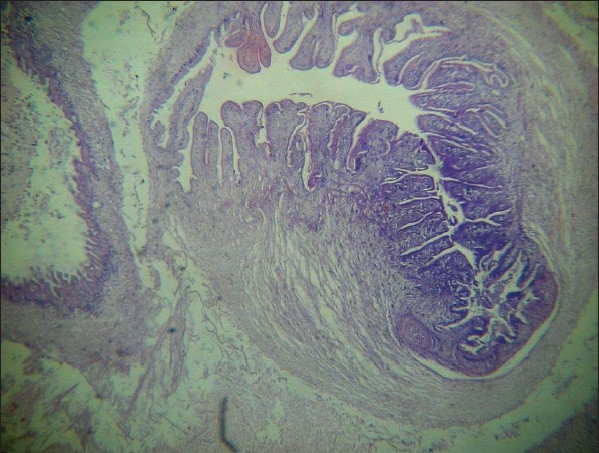
Histopathology of subcutaneous nodule showing cysticercosis (H and E, ×10)

Histopathological examination of a biopsied nodule showed evidence of cysticerci [Figure 4].

Colour Doppler study of the venous system of both lower limbs showed multiple small cystic nodular lesions, dispersed throughout the calf muscles and deltoid muscles with echogenic mural component, suggestive of scolex of *Taenia solium*.

Ultrasonography (USG) of both eyes revealed multiple small lesions in retro orbital tissue and along the lateral rectus muscle. In addition, there was posterior vitreous detachment in the right eye. Doppler study of both eyes was normal.

Evidence of cysticerci scattered in the brain parenchyma was seen on computerized tomography (CT) scan of the brain. Thyroid tests were normal.

Skin biopsy of a subcutaneous nodule on histopathological examinations revealed classical features of cysticercosis [Figure 4].

## Discussion

Generalized involvement of the body with cysticerci can affect the muscles. Clinically, the presentations can be of the myalgic type, nodular type or the uncommon pseudohypertrophic type.[1] ‘Disseminated muscular cysticercosis syndrome’[1] results when muscular pseudohypertrophy is often present with palpable subcutaneous nodules and seizures with abnormal mentation. Reports of such cases are rare. Muscle hypertrophy is usually asymptomatic in this pseudohypertrophic type and the affected muscles are generally nontender. They must be differentiated from pseudohypertrophy, muscular dystrophy, myotonia congenita, trichinosis, hypothyroidism, amyloidosis and glycogenesis of Type1 (Pompe's disease).[2] Diagnosis of cysticercosis involving the muscles is difficult clinically. Cysts which reside in the muscles are difficult to palpate, as they are often deep seated and numerous cysts lying side by side intramuscularly impart a smooth, shiny and tense appearance to the muscles. Ultrasonography is important in diagnosing the presence of cysticerci in these hypertrophied muscles, through revealing cystic lesions with or without calcification. Electromyography can be a useful tool in the diagnosis of muscle cysticercosis. Short duration, low amplitude motor unit potentials are the usual findings.[3] Symmetric painless enlargement of muscles, with seizures and subcutaneous nodules, in a case of generalized cysticercosis, can also be confirmed by muscle biopsy, which usually shows densely packed cysticerci in the muscles.[4] Pathogenesis of muscular hypertrophy in cysticercosis has not been clearly understood. It has been suggested that the dead larva may act as an irritant to the muscles, causing the inflammatory changes; and, response to steroids has raised the hypothesis that it may be a consequence of an allergenic response.

The involvement of the nervous system by cysticerci is known as neurocysticercosis (NCC). It is not uncommon in cases of disseminated cysticercosis. Neurocysticercosis can present with a wide range of manifestations like convulsions and signs and symptoms of a space-occupying lesion. Blurring of vision, often reported by these patients, can be due to a space occupying lesion (SOL) or papilledema caused by the cyst. At times, the NCC may be asymptomatic, if there is no pericystic inflammation.

To diagnose cases of neurocysticercosis, CT scans of the brain, with both contrast and non contrast studies, are essential. Wadia *et al*.[5] were the first to underline the importance of CT scan findings in disseminated cysticercosis of the central nervous system and the muscles. A relative lack of localizing signs and absence of any manifestation of raised intracranial tension marked their cases. The CT scan of the brain, however, revealed numerous small discrete lesions, which, on magnification, showed them to be scolices within cysticerci. Careful histopathology of a subcutaneous skin nodule when present with manifestations of neurocysticercosis can demonstrate the scolices of the parasite and greatly aid in the diagnosis.[6]

Oral cysticercosis can rarely be another component of disseminated cysticercosis. Dixon and Lipscomb[7] examined 450 cases of cysticercosis and found oral involvement in only 1.8% of the cases. Authors from a dental school in Brazil,[8] reported a rare and asymptomatic intraoral nodule in the right buccal mucosa of a 7-year-old boy. Biopsy of the nodule proved the diagnosis as cysticercosis.

Ocular involvement can be a part of the disseminated cysticercosis syndrome. Severe pain, blurring of vision and loss of vision are the common manifestations. Fundoscopic examination, ultrasonography of the eyes and Colour Doppler studies are useful in diagnosing ocular cysticercosis. Oral albendazole with corticosteroids can be useful in limiting persistent inflammation and improving ocular motility.[9] Surgical interference may be needed to remove orbital cysts in some cases.

A study from Rohtak, in India,[10] described a case of painless symmetrical and generalized muscular pseudohypertrophy due to cysticercosis, not unlike our case. The central nervous system and the eyes were spared in that case.

Cases of extensive cysticercosis, which had affected the brain, tongue and orbits and with huge, widespread hypertrophy of muscles, are rare and carry a sinister prognosis.
